# Translation, psychometric assessment, and adaptation of the state empathy scale among healthcare students

**DOI:** 10.1186/s12909-026-08645-6

**Published:** 2026-01-29

**Authors:** Inger Hilde Hagen, Vigdis Schnell Husby, Kjersti Natvig Antonsen, Randi Stokke, Elise Kvalsund Bårdsgjerde, Lars Aune Svarthaug, Marit Stuedahl, Susan Saga

**Affiliations:** 1 Department of Health Sciences in Ålesund, Norwegian University of Science and Technology NTNU, Postbox 1517, Aalesund, 6025 Norway; 2 Department of Health Sciences in Ålesund, Ålesund, Norway and Orthopaedic Department, Norwegian University of Science and Technology NTNU, Trondheim University Hospital, Post-box 3250 Torgarden, Trondheim, 7006 Norway; 3 Department of Public Health and Nursing, Norwegian University of Science and Technology NTNU, Trondheim, Norway; 4 Department of Health Sciences in Gjøvik, Norwegian University of Science and Technology NTNU, Postbox 191, Gjøvik, 2802 Norway

**Keywords:** Confirmatory factor analysis, State empathy scale, Validation, Healthcare students, Psychometric evaluation, Translation, Adaptation

## Abstract

**Background:**

Understanding patients’ experiences, opinions, and feelings is essential for health professionals to assess patients’ real needs and provide optimal treatment. Teaching and assessing healthcare students’ empathy is challenging in an educational context. The English measurement instrument State Empathy Scale, based on the theory of empathy, measures state empathy during message processing and was found to be suitable for measuring state empathy among healthcare students.

**Methods:**

This study aimed to translate and adapt the State Empathy Scale into Norwegian and assess its psychometric properties among healthcare students. The study was conducted among healthcare students at the Norwegian University of Science and Technology (NTNU). Participants consisted of 189 students from various healthcare study programs who took part in a VR 360° video learning design conducted during Trauma Week.

The translation process followed international guidelines and included independent forward translation, synthesis, back-translation, and expert committee review. Minor adaptations were made to tailor the scale to the healthcare education context. The revised version was field-tested among healthcare students and assessed using exploratory and confirmatory factor analysis.

**Results:**

Small adjustments to a healthcare student context were made, and the measurement instrument demonstrated good face/content validity. The Norwegian version of the State Empathy Scale’s structural validity was evaluated. It supported a three-factor structure similar to the original English instrument but with three removed items. The measurement instrument demonstrated good convergent and discriminant validity and internal consistency.

**Conclusions:**

The translated, adapted, and validated State Empathy Scale 9 - Norwegian version (SES9-No) is reliable and valid for assessing empathy among Norwegian healthcare students. This tool can facilitate the development and evaluation of empathy training programs, ultimately enhancing the quality of patient care.

**Supplementary Information:**

The online version contains supplementary material available at 10.1186/s12909-026-08645-6.

## Introduction

Clinical healthcare professionals are in regular contact with patients’ experiences of uncertainty, pain, and anxiety. Understanding patients’ experiences, opinions, and feelings is essential to assessing their needs and giving them optimal treatment. Hence, developing empathic skills is necessary [1]. Empathy is understanding other people’s feelings, what they mean, and how they convey these emotions to others. The concept encompasses multiple dimensions, incorporating both emotional and cognitive empathy [2]. Empathy is essential to the quality of a therapeutic care relationship [3].

Teaching and assessing students’ empathy is challenging. A systematic review concluded that the best way for nursing students to develop empathy is through clinical simulation focusing on disadvantaged patient groups and promoting reflective thinking [4]. In our study, we sought an instrument to measure healthcare students’ empathy after observing a patient’s experiences in a trauma bay through a virtual reality (VR) head-mounted display. There are limited assessment tools validated for use in assessing student empathy. Most researchers agree that empathy is to feel what someone else is feeling because of something that happened to them. The emotions of others are based on how we evaluate our situations [5]. Despite empathy being essential in healthcare, existing methods for assessing empathy among healthcare students, particularly in realistic or simulated contexts remain limited and inadequately validated.

The State Empathy Scale, developed by Shen in 2010 [6], is a measurement tool grounded in empathy theory that assesses state empathy during the process of message interpretation [7–14]. This measurement instrument was found to be adequate for our purpose. The tool can be understood as a process encompassing three dimensions: affective empathy, cognitive empathy, and associative empathy [6]. Affective empathy is triggered when individuals experience emotional responses to others, involving the understanding and sharing of their feelings. Cognitive empathy, on the other hand, pertains to perspective-taking, which includes recognizing, understanding, and adopting another person’s viewpoint. The third component is associative empathy, which relates to identification, social bonding, and relationship development [6]. The State Empathy Scale (SES**)** is particularly suited for medical simulation settings because it measures situational empathy—the immediate emotional and cognitive response to a specific clinical scenario. Unlike the Jefferson Scale of Empathy (JSE) [15] and Kiersma-Chen Empathy Scale (KCES) [16], which assess more stable, trait-based empathy, SES captures short-term changes in empathic engagement, making it ideal for evaluating the immediate impact of simulation exercises.

Our study aims to translate the SES into Norwegian, adapt it to a VR 360° video viewing in a healthcare context, and investigate its psychometric properties. More specifically, we investigated face/content validity, structural validity, convergent and discriminant validity of the factors, as well as the reliability of the scale.

## Method

### Design

This was a cross-sectional study aimed to translate the SES into Norwegian and assess its validity and reliability in a Norwegian sample of healthcare students. Guidelines for developing, translating, and validating a questionnaire [17] and the Consensus-based Standards for the selection of health Measurement Instruments (COSMIN) Checklist for evaluating the methodological quality of instrument properties were used [18].

### Participants and setting

The study was conducted at the Norwegian University of Science and Technology (NTNU) in January 2024. The study population consisted of students in bachelor’s programs in paramedic and radiography science and master’s programs in nurse anesthesia, pediatric nursing, intensive care nursing, and operating room nursing.

The students participated in a VR 360° video learning design created as part of the ‘Across Fjords and Mountains’ project [Norwegian: Fjord og fjell – NTNU]. In this project, the students participated in a Trauma Week featuring diverse trauma-related learning activities. The VR 360° video learning design aimed to provide reflection and insights into trauma patient care by taking the patient’s perspective in the VR trauma bay and reflecting together on the experience during the debriefing phase of the simulation training. The inclusion criteria were being a student participating in this VR 360° video learning design during Trauma Week at NTNU. When conducting factor analysis, it is generally recommended to have a sample size that is five to ten times larger than the number of items on the instrument [19]. The original State Empathy Scale has 12 items. Hence, the sample size for conducting Confirmatory factor analysis (CFA) can range from 60 to 120.

The students were asked to anonymously and digitally respond to the translated and adapted version of the State Empathy Scale accessed through a distributed QR code, using their mobile phones directly after completing the learning activity and before they left the room. Participation was voluntary, and by submitting their responses, participants consented to take part in the study.

### The measurement instrument

The State Empathy Scale, developed by Shen [6], was based on items from several existing scales. This self-report rating scale determines the levels of situational empathy students experience. The instrument is a 12-item scale, where four items are related to affective empathy (item 1 to item 4), four items are related to cognitive empathy (item 5 to item 8), and four items are related to associative empathy (item 9 to item 12), as illustrated in Table 1.

**Table 1:** Items and dimensions of the original state empathy scale by Shen [6]

Dimensions	Items
Affective Empathy	1. The character’s emotions are genuine. 2. I experienced the same emotions as the character when watching this message. 3. I was in a similar emotional state as the character when watching this message. 4. I can feel the character’s emotions.
Cognitive Empathy	5. I can see the character’s point of view. 6. I recognize the character’s situation. 7. I can understand what the character was going through in the message. 8. The character’s reactions to the situation are understandable.
Associative Empathy	9. When watching the message, I was fully absorbed. 10. I can relate to what the character was going through in the message. 11. I can identify with the situation described in the message. 12. I can identify with the characters in the message

**Fig. 1:**
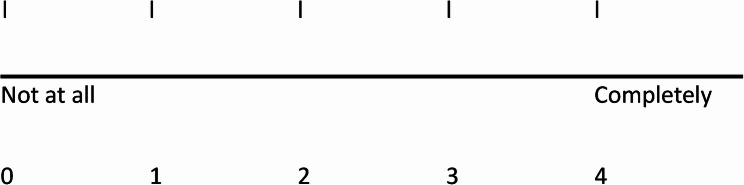
Visual representation of response options for the state empathy scale

The questions were measured on a 5-point Likert scale anchored at both poles (0= “not at all” and 4= “completely”) (Fig. 1). The original scale showed good internal consistency and discriminant and convergent validity [6].

### Translation process

After obtaining permission from the developer to translate the original State Empathy Scale into Norwegian and validate the instrument, the cross-cultural adaption process was employed [20] to generate a valid and reliable translation.

Initially, the word “character” was changed to “patient” and the word “message” was changed to “360° video” to better suit the instrument’s purpose. After that, three researchers (KNA, VSH, IHH) all fluent in English, independently translated the instrument from English into Norwegian. ChatGPT-4 [21] was then used to generate an alternative, fourth translation with the aim of providing additional linguistic suggestions. This machine-generated translation served as a supplement in the translation process. All four translations were compared and evaluated by the researchers before they were synthesized into a new version. Finally, KNA and VHS discussed the latest translated version and agreed upon the final version by consensus.

The Norwegian version was back-translated into English by two independent translators: One professional translator and one bilingual university employee blinded from the instrument’s original version. Finally, an expert committee consisting of faculty members compared the linguistic equivalence and cultural relevancy of the back-translated version of the instrument with the original version and reached a consensus on the translation of the State Empathy Scale. Thereafter, the back-translated version was reviewed by the developer of the original instrument, Lijiang Shen. Shen accepted the back-translation but suggested removing “360°” and replacing the word “video” with “media” to make the questionnaire more generic. We removed “360°” from “360° video” to broaden the applicability of the adapted instrument, ensuring it could be used with all forms of video content. However, media, as a broad term, includes various forms of communication such as text, images, audio, and video. We retained “video”, as our focus was to measure state empathy in students more specifically after experiencing the video, used as an educational tool.

Response options identical to the Likert scale used in the original instrument were employed in the translation. Next, the Norwegian version of the State Empathy Scale was field-tested in the target group. To ensure the translated scale’s content validity, the instrument was tested among five VR 360° video learning design facilitators and ten students from the intensive care nursing and operating room nursing master’s degree study program. The facilitators and students were invited by email to give feedback question-by-question on the instrument’s readability and comprehension.

### Analysis

IBM SPSS version 29 (IBM SPSS Statistics) was used to conduct descriptive statistics and exploratory factor analysis, while STATA 18.0 (StataCorp, Texas, US) was utilized for confirmatory factor analysis, convergent validity, discriminant validity, and composite reliability. There were no missing data because the electronic questionnaire required mandatory responses. The research team assessed content and linguistic validity through the instrument’s translation process and communication with the original developer. Additionally, the instrument was reviewed question-by-question by students and facilitators who had participated in the 360° VR videos during Trauma Week. To evaluate structural validity, exploratory factor analysis (EFA), with Oblimin rotation and confirmatory factor analysis (CFA) were utilized [17, 22]. The suitability of the data for factor analysis was assessed using the Kaiser-Meyer-Olkin (KMO) coefficient and Bartlett’s test of sphericity. A KMO coefficient above ≥ 0.60/0.70 and a statistically significant chi-square value from Bartlett’s test indicated that the data were appropriate for factor analysis [23].

EFA relies on item correlations, grouping items that have high correlations into a single factor. Each factor represents items that share variance explained by an underlying dimension. EFA aims to identify the variance of factors within the dimensions and to explain this variance as thoroughly as possible using the fewest number of factors [24]. We wanted to test if the item correlated with the same factors as in the original questionnaire by Shen.

CFA is a hypothesis-driven method used to test predefined hypotheses about factor structures [13–15]. We aimed to evaluate the factor structure proposed by Shen [6]. Analyses were conducted using Stata’s SEM procedure, and a Satorra–Bentler correction was applied to adjust for slight non-normality in the factor distribution, ensuring robust estimation of standard errors and fit indices. Our analysis assessed the covariance captured by the factors and evaluated how well the model fit the observed data. In Confirmatory Factor Analysis (CFA), an item with a high factor loading (ideally greater than 0.4) indicates that it shares a common variance with the latent factor [25, 26]. A variety of fit indices were utilized to evaluate the relationship between the observed data and the model developed by Shen [6]. Specifically, a χ²/degrees of freedom (df) ratio of ≤ 2 was considered a good fit, while a ratio of ≤ 3 was deemed acceptable [26]. Skewness and kurtosis were observed in the latent factor “Cognitive” during the normality assumption inspection. Consequently, the Satorra–Bentler-corrected χ² was employed in the analysis, as it is recommended for evaluating non-normal continuous endogenous variables [25].

Additionally, the following indices were utilized: the root mean square error of approximation (RMSEA) (≤ 0.05 represents a good fit, ≤ 0.10 is acceptable), the standardized root mean square residual (SRMR) (≤ 0.05 represents a good fit, ≤ 0.10 is acceptable), the comparative fit index (CFI) (≥ 0.95 represents a good fit, ≥ 0.90 is acceptable), and the Tucker–Lewis index (TLI) (≥ 0.95 represents a good fit, ≥ 0.90 is acceptable) [27]. The optimal model was chosen by thoroughly evaluating model fit, parameter estimates, and modification indices (MI). MI is an estimate of how much the chi-squared will be reduced if we estimate a particular extra parameter. A change based on MIs should, however, only be made if the MI is substantial and if there is a theoretical justification [28]. Although Likert-type items are ordinal, simulation studies suggest that robust ML performs adequately when items have five categories and sample size is moderate [29]. We therefore estimated the CFA using ML with Satorra-Bentler correction for non-normality. This approach is widely accepted for ordinal data under these conditions.

Evaluating the dimensionality of the questionnaire includes assessing internal consistency, which was measured using composite reliability (ρc) [27, 30]. Cronbach’s alpha (α) coefficients were calculated to facilitate comparisons with other studies. Values ≥ 0.7 are considered good for both composite reliability coefficients and Cronbach’s alpha (*α*) [24, 27]. Convergent and discriminant validity were assessed using squared correlations (SC) between factors and average variances extracted (AVE). An AVE greater than or equal to SC indicates no problems with discriminant validity, while an AVE of at least 0.5 suggests no issues with convergent validity [27]. To further evaluate the scale’s discriminant validity, additional indices were calculated. The Heterotrait–Monotrait ratio (HTMT) was estimated using Spearman correlations among the ordinal items, where values below 0.85 indicate adequate discriminant validity [31]. In addition, Maximum Shared Variance (MSV) and Average Shared Variance (ASV) were computed from squared correlations between composite factor scores, and discriminant validity was considered acceptable when MSV was lower than the AVE value [32, 33].

## Results

In this study, 211 students were invited to participate in the field test, and 189 (89.57%) responded to the State Empathy Scale. However, demographic characteristics of the 211 participants were not obtained due to the small sample sizes in some of the study programs.

### Content validity

As a part of the validation process, we used students and facilitators to comment on the instrument to assess the items’ relevance, readability, and equivalence. The relevance of the items was assessed by judging whether the purpose of measuring empathy by healthcare students was accomplished using VR. The students were asked to comment on each of the translated and modified items in the scale to assess their relevance and whether the questions were understandable (Table 1). Several respondents reported that item 1 was difficult to comprehend *(“The patient`s emotions are genuine*”). Comments like “*What are you asking?”* and “*I can only know what I feel*,* not the patient*” were recurring. Some students commented that items 2 and 3 were quite similar *(“I experienced the same emotions as the patient when I watched the video” and “I was in a similar emotional state as the patient when watching the video”).*

### Structural validity

Structural validity relates to the degree to which the instrument is an adequate reflection of the dimensionality of the construct to be measured (Mokkink et al., 2010b) [27]. We tested the three-dimensional structure involving 12 items developed by Shen [6]. The KMO statistics yielded a value of 0.827, and Bartlett`s test of sphericity had a value of *p* < 0.001, which was deemed sufficient. In the EFA analysis of the State Empathy Scale’s 12 questions, three factors were extracted, which explained 64% of the variance (Table 2). Although the three factors had some similarities with the original factor structure, several items (item 1, 4, 5, 6, 7,8 and 9) loaded on another factor than in the original factor structure proposed by Shen [6], and the EFA demonstrated cross-loadings (item 1 and item 7) with all factor loadings > 0.3, which revealed an uncertain factor structure [27].

**Table 2:** EFA-derived factor structure (Oblim rotation), translated state empathy scale’s 12 items, *n* = 189

Item number	Item	Factor loading on factor 1	Factor loading on factor 2	Factor loading on factor 3
11	I can identify with the situation described in the video	0.872		
10	I can relate to what the patient was going through in the video	0.814		
12	I can identify with the patient in the video	0.780		
3	I was in a similar emotional state as the patient when I watched the video		0.878	
2	I experienced the same emotions as the patient when I watched the video		0.786	
4	I can feel the patient`s emotion			0.722
9	When I watched the video, I was fully absorbed.			0.453
6	I recognize the patient`s situation.		0.821	
8	The patient’s reactions to the situation are understandable		0.786	
5	I can see the patient`s point of view.		0.643	
7	I can understand what the patient was going through in the video	0.362	0.613	
1	The patient`s emotions are genuine.	− 0.386	0.421	0.487

Due to the uncertain factor structure with cross-loadings revealed by the EFA, and because conclusions should not be based solely on EFA, we conducted a CFA. In CFA, the original three-factor solution proposed by Shen [6] was tested, and the starting model, Model 1, involved all 12 items and three factors. This model revealed standardized factor loadings (λ) of 0.38–0.86.38.86, with squared multiple correlations (R^2^) of 0.14–0.73.14.73. We revealed a poor fit: Satorra-Bentler *X*^2^ = 153.902 (*df 51)*,* X*^2^/*df* = 3.02, *p* < 0.001, RMSEA = 0.104, *p* for test of close fit = < 0.001, CFI = 0.87, TLI = 0.83, SRMR = 0.097. This indicated misspecification. Reliability assessed with the composite reliability coefficient (ρ_c_) was good for all three dimensions (Table 3). Scrutinizing factor loadings and residuals revealed no significant residuals, but other possible reasons for poor model fit. Two-factor loadings were < 0.4, where item 9 had the lowest loading (λ = 0.38), and also the lowest R^2^ -value (0.14). Item 9 concerned the student’s level of absorption of the video, which may be an expression of immersion rather than associative empathy which the factor measures.

**Table 3:** Goodness of fit measures for Model-1–5, *n* = 189

Fit measure	Model-1 3 factors 12 items	Model-2 3 factors 11 items (item 9 removed)	Model-3 3 factors 10 items (items 9 and 1 removed)	Model-4 3 factors 9 items (items 9, 1 and 7 removed)	Model-5 3 factors 9 items (items 9, 1 and 7 removed) Cov(e.x2, e.x4)
*χ* ^2^	153.902	122.931	76.307	53.649	42.761
*p*-value	< 0.001	< 0.001	< 0.001	< 0.001	< 0.001
*χ*^2^/df	3.02	3.00	2.38	2.24	1.86
RMSEA	0.104	0.103	0.086	0.081	0.068
*p*-value (close fit test)	< 0.001	< 0.001	< 0.001	0.014	0.082
SRMR	0.097	0.084	0.069	0.060	0.057
CFI	0.87	0.89	0.94	0.95	0.97
TLI	0.83	0.86	0.91	0.93	0.95
$\rho c = \frac{\left(\sum \lambda\right)^{2}}{\left(\sum \lambda\right)^{2} + \sum (\uptheta)}$	0.76–0.84	0.76–0.88	0.76–088.76	0.71–0.88	0.71–0.88

*Χ*^2^/df (≤ 2 good fit, ≤ 3 acceptable). RMSEA; root mean square error of approximation (≤ 0.05 good fit, ≤ 0.10 acceptable). SRMR; standardized root mean square residuals (≤ 0.05 good fit, ≤ 0.10 acceptable), CFI; the comparative fit index (≥ 0.95 good fit, ≥ 0.90 acceptable). TLI; Tucker-Lewis index (≥ 0.95 good fit, ≥ 0.90 acceptable). *ρc*; composite reliability (*ρc* ≥ 0.7 good)

Thus, item 9 was removed, and we ran CFA again. This solution, termed Model-2, with 11 items revealed only a slightly improved fit: Satorra-Bentler *X*^2^ = 122.931 (*df 51)*,* X*^2^/*df* = 3.00, *p* < 0.001, RMSEA = 0.103, *p* for test of close fit = < 0.001, CFI = 0.89, TLI = 0.86, SRMR = 0.084. This indicated misspecification as well. In this model, item 1 had a factor loading of < 0.4 and the lowest R^2^-value (0.16). Several students commented that they found it hard to give a proper response to this question about “whether the patient’s feelings in the video were real or not”.

Therefore, item 1 was removed, and we ran CFA once more, giving Model-3 (10 items included) with an improved fit: Satorra-Bentler *X*^2^ = 76.307 (*df 32)*,* X*^2^/*df* = 2.38, *p* < 0.001, RMSEA = 0.086, *p* for test of close fit = < 0.001, CFI = 0.94, TLI = 0.91, SRMR = 0.069.

We found however that item 7 cross-loaded on all three factors and, therefore, seemed unreliable. Item 7 deals with whether students can understand what the patient went through when they watched the VR 360° video, which clearly can load on both Cognitive empathy, Affective empathy, and Associative empathy. Although neither students nor facilitators had any comments on this item, the research team found it reasonable that this item involves both feelings (Affective) and understanding (Cognitive) and that watching a trauma admission through the patient’s perspective involves identification with the patient (Associative). Since this item did not belong to only one factor, the research team decided to remove it.

Removing item 7 from the model led to Model-4, consisting of 9 items, which revealed an improved fit: Satorra-Bentler *X*^2^ = 53.649 (*df 24)*,* X*^2^/*df* = 2.24, *p* < 0.001, RMSEA = 0.081, *p* for test of close fit = 0.014, CFI = 0.95, TLI = 0.93, SRMR = 0.06.

Modification indices indicated several possible changes for our model. Allowing the error terms for a pair of items to be correlated has a substantial modification index value and makes conceptual sense. The pair of items 5 and 6 demonstrated an MI = 10.59, and we tried to include a correlated error term between them. However, this did not improve the fit and was not included in the model. The pair of items 2 and 4 had MI of 9.30, and we included a correlated error term between them, which improved the model substantially in Model-5: Satorra-Bentler *X*^2^ = 42.761 (*df 23)*,* X*^2^/*df* = 1.86, *p* < 0.001, RMSEA = 0.068, *p* for test of close fit = 0.082, CFI = 0.97, TLI = 0.95, SRMR = 0.057. During model refinement, allowing a correlated error between Item 2 (“I experienced the same emotions as the patient when I watched the video”) and Item 4 (“I can feel the patient’s emotion”) improved overall model fit. This modification was theoretically justified because both items reflect closely related aspects of affective empathy, emotional mirroring and empathic resonance, which are conceptually linked in the underlying construct. Although the items share semantic similarities, they capture distinct nuances of the empathy experience and contribute meaningfully to the factor, as indicated by their strong loadings (0.89, and 0.92). Removing one of these items would reduce the factor to only two indicators, which is generally not recommended for confirmatory factor analysis due to identification and reliability concerns. Therefore, both items were retained to preserve the integrity and conceptual coverage of the Affective Empathy factor. Full standardized loadings and R² values for all intermediate models (Models 1–5) are provided in Supplementary file S1.

Discriminant validity was confirmed with all AVE values ≥ SC. However, we found problems with convergent validity where the AVE-value in the Cognitive factor was 0.463 and thereby slightly < 0.5, while Affective (0.567) and Associative (0.714) factors were confirmed. Discriminant validity was further evaluated using HTMT, MSV, and ASV. All HTMT values were well below the recommended threshold of 0.85 (Affective–Cognitive = 0.43; Affective–Associative = 0.57; Cognitive–Associative = 0.38), supporting adequate discriminant validity. MSV values were lower than the AVE for all three factors, indicating that each factor shared less variance with other constructs than with its own items. MSV/ASV values were 0.218/0.156 (Affective), 0.095/0.089 (Cognitive), and 0.218/0.150 (Associative). These results confirm that the three SES9-No factors remain empirically distinct.

After modifying the instrument through the CFA analyses, we ran an EFA again with nine items. Three factors were also extracted this time, which explained 73% of the variance. The Rotated Component Matrix shows the items loading on the same factors as the original version. The factor loadings were high, between 0.71 and 0.89.

**Fig. 2:**
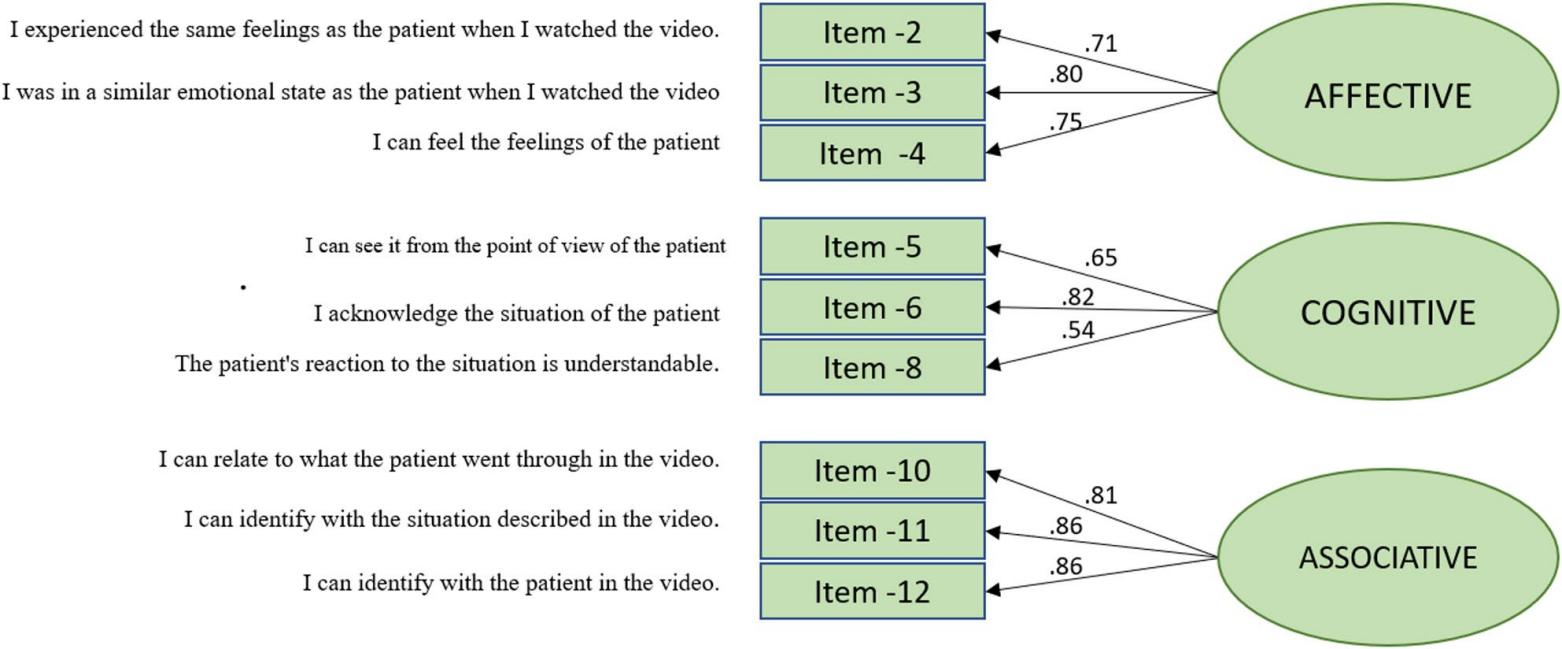
The best fitting measurement model of the Norwegian version of the State Empathy Scale (SES9-No)

The total Cronbach’s alpha for all nine items was 0.82. In the corrected item-total correlation, the items correlated between 0.31 and 0.66. The total correlation for the Affective dimension was (0.79), for the Cognitive dimension (0.69), and the Associative dimension (0.88). Model 5 of the State Empathy Scale, with 3 factors and 9 items, provided the best model with a good fit (Fig. 2; Table 4).

**Table 4:** Model-5^a^: the best fitting measurement model including standardized factor loadings, t-values, and squared multiple correlations, *n* = 189

Item	Parameter		Stata Estimate^b^	t-value^c^		*R* ^2d^
*Affective*						
*Item-2*	λx 2,1		0.89	11.65		0.51
*Item-3*	λx 3,1		0.66	10.37		0.64
*Item-4*	λx 4,1		0.92	12.77		0.56
*Cognitive*						
*Item-5*	λx 5,2		0.65	9.24		0.42
*Item-6*	λx 6,2		0.81	12.71		0.67
*Item-8*	λx 8,2		0.55	6.98		0.30
*Associative*						
*Item-10*	λx 10,3		0.81	25.69		0.65
*Item-11*	λx 11,3		0.86	24.38		0.74
*Item-12*	λx 12,3		0.86	28.02		0.75
*Factor*						
*ρ*_*c*_^*e*^_*AFFECTIVE*_			*ρ*_*c*_			0.80
*ρ*_*cCOGNITIVE*_			*ρ*_*c*_			0.71
*ρ*_*cASSOCIATIVE*_			*ρ*_*c*_			0.88

^a^Three-factor solution including 9 items (items 1, 7 and 9 are dismissed)

^b^Completely Standardized Factor Loadings

^c^Significant at the 1% level

^d^Bentler-Raykov squared multiple correlation coefficient

^e^Composite reliability $$\rho c = \frac{\left(\sum \lambda\right)^{2}}{\left(\sum \lambda\right)^{2} + \sum (\uptheta)}$$

## Discussion

The original State Empathy Scale consists of 12 items distributed into three factors. The instrument was slightly adapted into the context of VR simulation training in a healthcare educational context, translated into Norwegian, and psychometrically tested on 189 healthcare students. This study aimed to examine the psychometric properties of the Norwegian version of the State Empathy Scale by evaluating its face and content validity, structural validity, convergent validity, discriminant validity among the factors, and internal consistency. Through the translation process, the instrument was adapted linguistically, culturally, and contextually. Our study identified a revised three-factor solution consistent with the scale developed by Shen [6]. We named this version the State Empathy Scale-9 Revised Norwegian version (SES9-No) (Supplementary file 2 and Supplementary file 3).

Empathy plays an important role in establishing therapeutic relationships between healthcare professionals and patients [34] and can improve the quality of care [35]. Several instruments have been developed to measure empathy [36, 37]. The Kiersma-Chen Empathy Scale (KCES) has been widely used to assess the empathy of healthcare students [16, 38–40]. Adapting instruments to the current research setting is essential, and the KCES did not fit our purpose. We wanted to measure whether the students’ experienced empathy with a patient in a trauma bay setting using VR 360**°** video. To our knowledge, no instrument is specifically developed to assess empathy when the students experience the trauma bay from the patient’s view through VR 360**°** video. With some modifications, we found the State Empathy Scale to be the most suitable questionnaire for this purpose [6]. Therefore, this study aimed to translate them into Norwegian, slightly modify the items of the State Empathy Scale, investigate the tool’s psychometric properties, and determine whether revisions of our revised version were needed.

In our study, we investigated the instrument’s face/content and structural validity. The face/content validity of the instrument was assessed by students and facilitators, judging whether its 12 items were relevant for healthcare students using VR 360° video to develop empathy for the patient in a trauma bay setting. The translated measurement instrument was also adapted to an educational setting with the purpose of measuring healthcare students’ empathy in a trauma bay. The word “character” was changed to “patient” in all items that initially had the term “character” and the word “message” was changed to “video” in all items that initially had the term “message”. Structural validity concerns the instrument’s dimensionality of the construct to be measured [24]. In this study, we investigated the three dimensions of affective, cognitive, and associative as dimensions of Empathy, which are theory-based concepts [2, 6]. Some items indicated misspecification of the model; items 1 and 9 had low factor loadings, and item 7 cross-loaded on all factors. This was in line with some of the comments from students and facilitators regarding overlap and an unclear meaning of those items. Additionally, item 9 could be a confounding factor since it is about immersion, and this is a process where the audience/user is absorbed cognitively and affectively into the imagery presented in the video narrative [41]. This could involve the loss of senses of reality, time, and self. Therefore, the SES9-No is reduced to 9 items distributed in 3 factors (factor 1 covers the affective dimension, factor 2 covers the cognitive dimension, and factor 3 covers associative items). Although the AVE value for the Cognitive factor (0.463) was slightly below the recommended threshold, the factor demonstrated acceptable item loadings, satisfactory composite reliability, and strong discriminant validity. In line with current SEM recommendations, such patterns indicate that the factor shows adequate convergent validity despite the marginal AVE value [32, 33].

This study did not include formal quantitative assessments of content validity, such as I-CVI and S-CVI, which are strongly recommended in international guidelines like COSMIN. These indices provide a systematic and reproducible approach to evaluating expert consensus on item relevance, a core aspect of tool validation [42]. The absence of such quantitative measures limits the robustness of the validity evidence and restricts comparability with other validation efforts. Future research should incorporate these procedures from the outset to ensure methodological rigor and strengthen the instrument’s credibility for clinical and research use.

In a recent study aimed to measure empathy in master’s students in healthcare professions using a modified version of the State Empathy Scale (12 items) [43], they found that a single factor was the most straightforward conceptualization of the measure with good internal consistency (Cronbach’s alpha averaged 0.87 across three-time points). According to Keefer, a validated instrument that provides information regarding university students’ emotional development and contributes to the design and evaluation of intervention programs is needed [44]. The revised version of the State Empathy Scale has manifold educational applications, including assessing the emotional development needs that students may present at the beginning of their studies, determining students’ level of self-reported empathy when experiencing videos, and using it for educational purposes. Developing empathy is essential for healthcare students during their education. Understanding patients’ perspectives through VR 360° videos can help healthcare students better grasp what patients experience in various settings.

### Limitations and strengths

One of the strengths of this study was the comprehensive methodology used to translate [17], adapt, and validate a Norwegian version of the State Empathy Scale, in accordance with the COSMIN guidelines [18]. Furthermore, our study benefited from a large sample size and response rate with no missing data. Our study demonstrated good reliability in terms of internal consistency in the total scale (nine items) and the three subscales (three items in each). Cronbach’s alpha values in the original scale developed by Shen [6] were somewhat higher (2010), probably due to a higher number of included items. Nonetheless, certain limitations must be considered. The Norwegian version of the State Empathy Scale was not pilot-tested directly after the translation process. However, after the field-test had been performed and survey data was collected, a pilot-test (feedback on the instrument’s items from students and facilitators) gave valuable information about the instrument and its items. This information was used alongside CFA when the structural validation was investigated. The use of convenience sampling may limit the generalizability of the findings, as the sample may not adequately represent the broader population of healthcare students. Test-retest reliability was not assessed, which restricts the ability to evaluate the temporal stability of the measurement instrument. In this study, demographic variables such as age and gender were not collected. Another limitation is that the questionnaire was completed shortly after the students had participated in the VR 360° video learning design and thus might have been overwhelmed. On the other hand, answering the questionnaire much later could have resulted in recall bias. This study assessed key psychometric properties, including content validity, structural validity, and internal consistency, which were feasible within the cross-sectional design. However, other important measurement properties recommended by COSMIN, such as test–retest reliability, measurement error, responsiveness, and concurrent validity, could not be evaluated because they require repeated measurements or inclusion of external scales, which were not part of the original data collection. Content validity was tested among five VR 360° video learning design facilitators and ten students from the intensive care nursing and operating room nursing master’s degree study program. Content validity was not evaluated quantitatively by Item-level Content Validity Index (I-CVI) or Scale-level Content Validity (S-CVI). These limitations should be addressed in future research to strengthen evidence for temporal stability and construct validity, to meet international standards (COSMIN).

## Conclusion

The Norwegian version of the State Empathy Scale (SES9-No) was translated from English to Norwegian and adapted to the healthcare educational context. The findings show satisfactory face/content- and structural validity with three empathy subscales for healthcare students. The Norwegian version of the questionnaire demonstrated satisfactory evidence of reliability and validity for assessing state empathy among Norwegian healthcare students after viewing a VR 360° video from the patient’s perspective in a trauma bay setting.

## Supplementary Information

Supplementary Material 1.

Supplementary Material 2.

## Data Availability

The datasets used and/or analyzed during the current study are available from the corresponding author on reasonable request. Please note that all data are in Norwegian.

## Abbreviations

Abbreviation: Text
VR: Virtual reality
NTNU: Norwegian University of Science and Technology
CFA: Confirmatory factor analysis
EFA: Exploratory factor analysis
SES9-No: State Empathy Scale-9 Revised Norwegian version
RMSEA;: Root Mean Square Error of Approximation
SRMR;: Standardized Root Mean Square Residuals
I-CVI: Item-level Content Validity Index
S-CVI: Scale-level Content Validity Index
