# Supplementary material for: Translation, psychometric assessment, and adaptation of the state empathy scale among healthcare students
Source: BMC Med Educ. 2026 Jan 29;26:317. doi: 10.1186/s12909-026-08645-6 (PMC12918513; doi:10.1186/s12909-026-08645-6)
Supplement: Supplementary file 1 — Supplementary Material 1. [file 12909_2026_8645_MOESM1_ESM.docx]

Supplementary Files

**Supplementary Table S1. Standardized Factor Loadings and R² Values for All CFA Models**

This table presents standardized factor loadings (λ) and R² values for each item across the five CFA models tested during the model refinement process of the State Empathy Scale (SES). Items removed during model modification are marked with “–”.

|  | | Model 1 | | Model 2 | | Model 3 | | Model 4 | | Model 5 | |
| --- | --- | --- | --- | --- | --- | --- | --- | --- | --- | --- | --- |
|  |  | λ | R^2^ | λ | R^2^ | λ | R^2^ | λ | R^2^ | λ | R^2^ |
| Affective | Item-1 | 0.40 | 0.16 | 0.40 | 0.16 | - | - | - | - | - | - |
|  | Item-2 | 0.76 | 0.58 | 0.76 | 0.58 | 0.71 | 0.51 | 0.71 | 0.51 | 0.89 | 0.80 |
|  | Item-3 | 0.75 | 0.56 | 0.75 | 0.56 | 0.79 | 0.63 | 0.80 | 0.64 | 0.66 | 0.44 |
|  | Item-4 | 0.73 | 0.54 | 0.73 | 0.54 | 0.75 | 0.57 | 0.75 | 0.56 | 0.92 | 0.84 |
| Cognitive | Item-5 | 0.65 | 0.42 | 0.65 | 0.42 | 0.64 | 0.41 | 0.65 | 0.42 | 0.65 | 0.43 |
|  | Item-6 | 0.70 | 0.49 | 0.70 | 0.50 | 0.70 | 0.49 | 0.82 | 0.67 | 0.81 | 0.66 |
|  | Item-7 | 0.74 | 0.55 | 0.74 | 0.55 | 0.75 | 0.56 | - | - | - | - |
|  | Item-8 | 0.58 | 0.34 | 0.58 | 0.34 | 0.58 | 0.34 | 0.54 | 0.30 | 0.55 | 0.30 |
| Associative | Item-9 | 0.38 | 0.14 | - | - | - | - | - | - | - | - |
|  | Item-10 | 0.82 | 0.67 | 0.81 | 0.66 | 0.81 | 0.66 | 0.81 | 0.65 | 0.81 | 0.66 |
|  | Item-11 | 0.86 | 0.73 | 0.86 | 0.74 | 0.86 | 0.74 | 0.86 | 0.74 | 0.86 | 0.74 |
|  | Item-12 | 0.86 | 0.73 | 0.86 | 0.75 | 0.86 | 0.75 | 0.86 | 0.75 | 0.86 | 0.74 |
| **Note.** Model 1 = original 12‑item model; Model 2 = item 9 removed; Model 3 = items 9 and 1 removed; Model 4 = items 9, 1 and 7 removed; Model 5 = final model with correlated error terms between items 2 and 4.  λ =Standardized loadings (completely standardized solutions). | | | | | | | | | | | |

**Supplementary file 2.** Norwegian version (SES9-No) and the original Englich items of State Empathy Scale

| **Dimension** | **Item** | **Norwegian translation** | **English original version** |
| --- | --- | --- | --- |
| **Affective Empathy** | 1 | Jeg opplevde de samme følelsene som pasienten, da jeg så videoen. | I experienced the same emotions as the character when watching this message. |
|  | 2 | Jeg var i en lignende følelsesmessig tilstand som pasienten da jeg så videoen. | I was in a similar emotional state as the character when watching this message. |
|  | 3 | Jeg kan føle pasientens følelser. | I can feel the character’s emotions. |
| **Cognitive Empathy** | 4 | Jeg kan se det fra pasientens synspunkt. | I can see the character’s point of view. |
|  | 5 | Jeg anerkjenner pasientens situasjon. | I recognize the character’s situation~~.~~ |
|  | 6 | Pasientens reaksjon på situasjonen er forståelig. | The character’s reaction to the situation is understandable |
| **Associative Empathy** | 7 | Jeg kan relatere til det pasienten gjennomgikk i videoen. | I can relate to what the character was going through in the video. |
|  | 8 | Jeg kan identifisere meg med situasjonen som er beskrevet i videoen. | I can identify with the situation described in the message. |
|  | 9 | Jeg kan identifisere meg med pasienten i videoen. | I can identify with the characters in the message. |
| Note. The original English version also contain the following items:  ^a^The character’s emotions are genuine.  ^b^I can understand what the character was going through in the message.  ^c^When watching the message, I was fully absorbed. | | | |

**Supplementary file 3. Visual representation of response options to the original English version of State Empathy Scale and the Norwegian version (SES9-No)**

| **Value** | **0** | **1** | **2** | **3** | **4** |
| --- | --- | --- | --- | --- | --- |
| **English** |  | | | | |
|  | **Not at all** |  |  |  | **Completely** |
| **Norwegian** | **Ikke i det hele tatt** |  |  |  | **Fullstendig** |

Ref: Hagen et.al (2025)......

Note: Full reference to this article must be included on all typed versions of the SES9-No
