# Supplementary figures and images for: Translation, psychometric assessment, and adaptation of the state empathy scale among healthcare students
Source: BMC Med Educ. 2026 Jan 29;26:317. doi: 10.1186/s12909-026-08645-6 (PMC12918513; doi:10.1186/s12909-026-08645-6)

**Additional File**

File name: Graphic 1


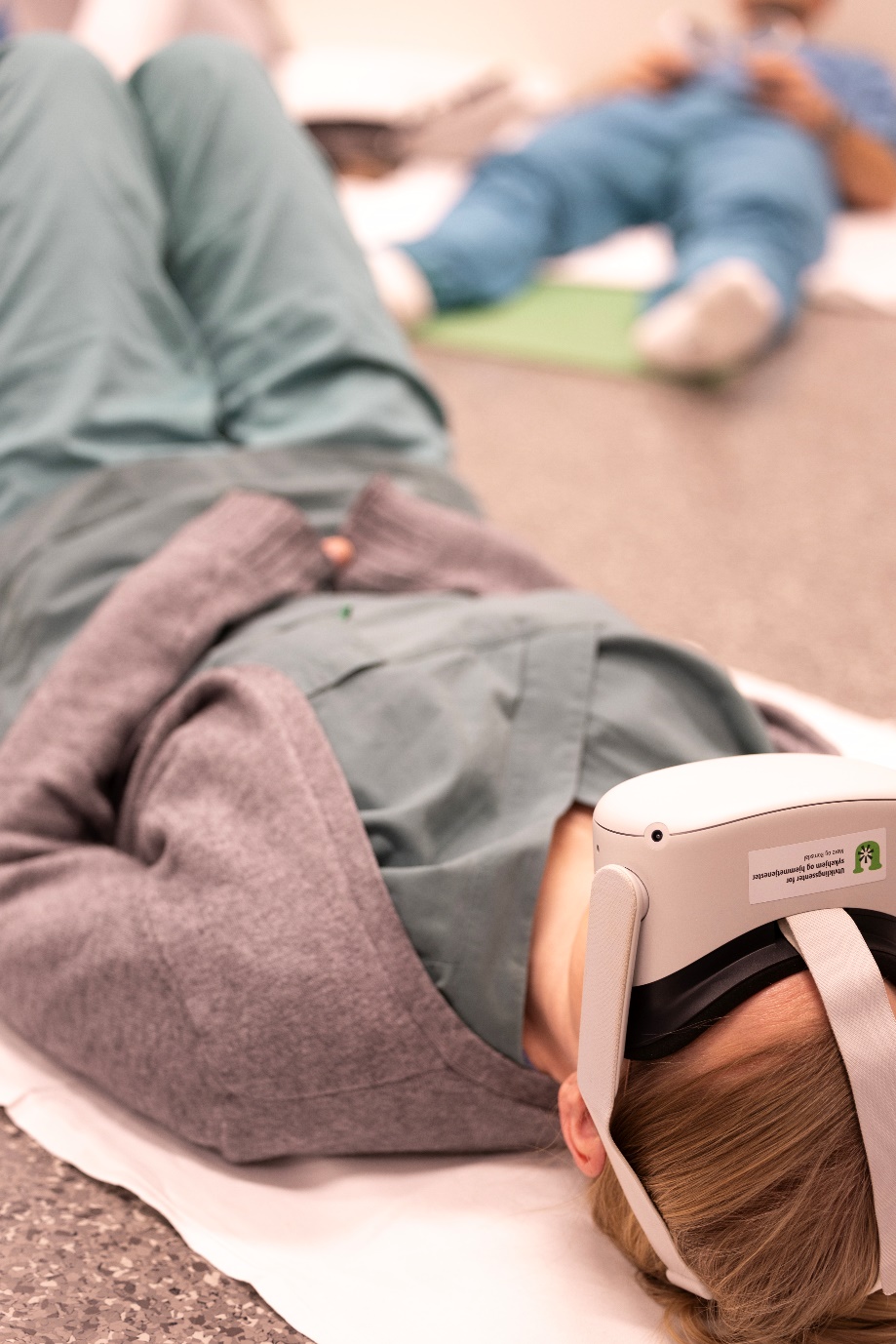

Supplement: Supplementary file 2 — Supplementary Material 2. [file 12909_2026_8645_MOESM2_ESM.docx]
